# 

**Published:** 1890-05

**Authors:** 


VOL. XI.] PUBLISHED MONTHLY AT THREE DOLLARS A YEAR. SINGLE COPIES, THIRTY CENTS. [No. 5.

### THE JOURNAL
OF
### COMPARATIVE MEDICINE
AND
### VETERINARY ARCHIVES.

EDITED BY

W. A. CONKLIN, Ph. D., D. V. S.,

*Director of Zoological Gardens, New York City.*

RUSH SHIPPEN HUIDEKOPER, M.D., Veterinarian, (*Alfort.*)

*Philadelphia.*

MAY, 1890.

A. L. HUMMEL, M.D., PUBLISHER,

Chestnut and Sixteenth Streets, Philadelphia, Pa.

LONDON: BALLIERE, TINDALL & COX.

